# N-Acetylcysteine Displaces Glutathionyl-Moieties from Hg^2+^ and MeHg^+^ to Form More Hydrophobic Complexes at Near-Physiological Conditions

**DOI:** 10.3390/molecules28196762

**Published:** 2023-09-22

**Authors:** Maryam Doroudian, Michelle E. Thibault, Jürgen Gailer

**Affiliations:** Department of Chemistry, University of Calgary, Calgary, AB T2N 1N4, Canada; maryam.doroudian@ucalgary.ca (M.D.); michelle.thibault@ucalgary.ca (M.E.T.)

**Keywords:** mercury (II), methylmercury, N-acetylcysteine, glutathione, ICP-AES, liquid chromatography, hepatocytes, mass spectrometry

## Abstract

The anthropogenic release of Hg is associated with an increased human exposure risk. Since Hg^2+^ and MeHg^+^ have a high affinity for thiols, their interaction with L-glutathione (GSH) within mammalian cells is fundamentally involved in their toxicological chemistry and excretion. To gain insight into the interaction of these mercurials with multiple small molecular weight thiols, we have investigated their competitive interactions with GSH and N-acetylcysteine (NAC) at near-physiological conditions, using a liquid chromatographic approach. This approach involved the injection of each mercurial onto a reversed-phase (RP)-HPLC column (37 °C) using a PBS buffer mobile phase containing 5.0 mM GSH to simulate cytosolic conditions with Hg being detected in the column effluent by an inductively coupled plasma atomic emission spectrometer (ICP-AES). When the 5.0 mM GSH mobile phase was amended with up to 10 mM NAC, gradually increasing retention times of both mercurials were observed. To explain this behavior, the experiment with 5.0 mM NAC and 5.0 mM GSH was replicated using 50 mM Tris buffer (pH 7.4), and the Hg-containing fractions were analyzed by electrospray ionization mass spectrometry. The results revealed the presence of Hg(GS)(NAC) and Hg(NAC)_2_ for Hg^2+^ and MeHg(GS) and MeHg(NAC) for MeHg^+^, which suggests that the coordination/displacement of GS-moieties from each mercurial by the more hydrophobic NAC can explain their retention behavior. Since the biotransformations of both mercurials were observed at near-physiological conditions, they are of toxicological relevance as they provide a biomolecular explanation for some results that were obtained when animals were administered with each mercurial and NAC.

## 1. Introduction

The consumption of unprecedented quantities of fossil fuels and mining activities are key components of industrialization processes, leading to the mobilization of considerable amounts of toxic metals from the earth’s crust into the global biosphere [1]. Owing to their ongoing dispersal into different environmental compartments, the chronic low-level exposure of humans to toxic metals, predominantly via food, is inevitable [2]. While mercury (Hg) compounds are present at low concentrations in environmental media, such as soil and water, their persistence and tendency to undergo bioaccumulation in the food chain pose a health risk to human populations [3]. The risk that is inherently associated with the chronic exposure of humans to Hg compounds was brought to worldwide attention during the Minamata disaster, which unfolded in Japan in the 1950s and involved the neurotoxin MeHg^+^ [4]. MeHg^+^ and Hg^2+^ represent the two most toxicologically important Hg species to which humans are chronically exposed [5]. Due to their distinct physicochemical characteristics, each of these mercurials exhibits different mechanisms of toxicity in mammals [6]. Since MeHg^+^ is one of the most potent neurotoxins known to man [7], extensive studies have been conducted to explore the effects of human exposure to this pollutant, revealing its tendency to accumulate in the central nervous system (CNS) at relatively high concentrations [8]. The bioaccumulation of MeHg^+^ in the CNS can result in neurotoxic effects not only in adults but also in the fetus [9], as this mercurial has also been demonstrated to cross the placental barrier in pregnant women. Conversely, chronic human exposure to Hg^2+^ is predominantly associated with nephrotoxicity [10] and hepatotoxicity [11].

**Figure 1:**
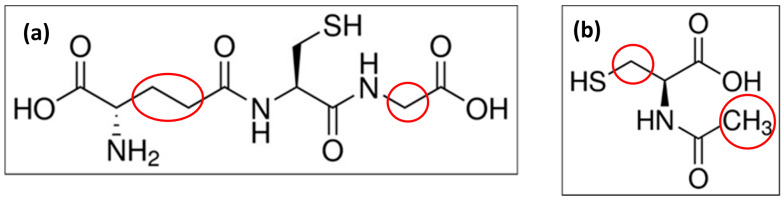
The molecular structure of L-glutathione (GSH) (**a**) and N-acetyl-L-cysteine (NAC) (**b**) with hydrophobic moieties highlighted in red. Note that the hydrophobic acetyl-moiety in NAC makes this thiol comparatively more hydrophobic as it is terminal, whereas the hydrophobic moieties in GSH are buried in its structure.

Owing to the high affinity of Hg^2+^ and MeHg^+^ for thiols, their metabolism in mammals fundamentally involves interactions with small molecular weight thiols [12], including the tripeptide L-glutathione (GSH, Figure 1a), which is present in all mammalian cells at concentrations up to 10 mM. Additionally, proteins containing thiol groups, such as human serum albumin [13] and hemoglobin [14], also play an important role in the mammalian metabolism of these mercurials [14]. In fact, investigations into the binding of Hg^2+^ and MeHg^+^ to intracellular thiols in intact human erythrocytes at 25 °C [~4–6 mM hemoglobin (Hb) and ~2.2 mM GSH] using ^1^H spin-echo NMR spectroscopy have revealed the intra-erythrocytic formation of GS-Hg-Hb [15] and MeHg-GS [16], with exchange lifetimes (between free and bound GS-ligand in each complex) of ~30 s [15] and ~0.01 s [16], respectively. The importance of GSH in the metabolism of these mercurials is further substantiated by the fact that in rats, both Hg^2+^ and MeHg^+^ are excreted into the bile as GS-complexes [17,18]. Furthermore, the most efficient approved antidotes for the treatment of animals [19] and humans [20] exposed to Hg^2+^ are the dithiols sodium 2,3-dimercapto-1-propanesulfonate (DMPS) and meso-2,3-dimercaptosuccinic acid (DMSA) [21], which hasten its urinary excretion due to the in vivo formation of water-soluble Hg complexes [22]. However, since these agents also exhibit a tendency to mobilize essential metals [23], there is an urgent need to develop more potent antidotes that are more selective for the targeted metal and that are associated with minimal or, ideally, no toxic side effects [24].

N-acetylcysteine (NAC, Figure 1b) is a sulfhydryl-containing antioxidant that can protect against MeHg^+^-induced embryotoxicity [25]. It can also affect the toxicity of both mercurials, even though the underlying biomolecular mechanisms are unknown [26]. There are, however, conflicting reports regarding the effectiveness of NAC against HgCl_2_ toxicity [10]. NAC contains a thiol (–SH) group, which serves as a potential coordination site for mercurials, but it also contains the rather hydrophobic acetyl group, which has been exploited to improve the HPLC separation of Hg^2+^ and MeHg^+^ based on increasing their hydrophobicity on RP-HPLC columns [27]. Unlike other chelating agents, NAC can act directly as a free radical scavenger and is converted in the body to metabolites that are capable of stimulating GSH synthesis [28]. While it is known that Hg^2+^ and/or MeHg^+^ will interact with GSH once they enter mammalian cells, a better understanding of these intracellular interactions, particularly when modified by exogenous thiol ligands, remains a fundamental bioinorganic chemistry problem with toxicological relevance. Therefore, there is a crucial need to probe the interaction of each of these mercurials with GSH and exogenous thiols, such as NAC, at near-physiological conditions. Understanding these interactions could provide new insight into their subsequent intracellular fate, such as the potential transport of each mercurial to specific organelles and/or the cell surface for their potential excretion. Once inside the cell cytosol, each mercurial competes for multiple ligands, including a vast number of cytosolic proteins. However, the intracellular concentration of endogenous GSH (5.0–7.5 mM range) contributes significantly to the formation of Hg-complexes, determining their biochemical fate in hepatocytes [18,29].

One method to investigate the binding of MeHg^+^ and Hg^2+^ to multiple thiol ligands involves a liquid chromatographic approach, which has previously provided insight into the competitive binding of each of these mercurials to GSH and L-cysteine (Cys) at near-physiological conditions [30]. In brief, a C_18_ reversed phase (RP) HPLC column was equilibrated with a mobile phase, the chemical composition of which simulated the cytosolic conditions of hepatocytes (i.e., 5.0 mM GSH in PBS buffer). Then each mercurial was injected into this RP-HPLC system, and the utilization of an inductively coupled plasma atomic emission spectrometer (ICP-AES) as a Hg-specific detector revealed a unique retention behavior of Hg^2+^ and MeHg^+^ with increasing Cys concentrations (0.5 and 1.0 mM steps up to 10 mM) in the 5.0 mM GSH-containing mobile phase. The observed decrease in retention time iof each mercurial was driven by two fundamental processes, namely (a) the on-column formation of progressively less hydrophobic Cys complexes and (b) the strength of the Hg-S bonds of the on-column formed Hg-species.

Since GSH is by far the most abundant small molecular weight thiol in mammalian cell cytosols, the aforementioned RP-HPLC-ICP-AES approach was employed to investigate changes in the coordination of each mercurial to GSH with increasing NAC concentrations at near-physiological conditions in the absence of cytosolic proteins. The retention behavior of Hg^2+^ and MeHg^+^ was observed as a function of increasing NAC concentrations while maintaining a constant mobile phase concentration of 5.0 mM GSH in PBS buffer. To rationalize the observed retention behavior for each mercurial in terms of the bioinorganic chemistry that unfolded on the column, the experiment was repeated with specific mobile phase scenarios using a mobile phase containing Tris buffer (pH 7.4). This allowed us to collect fractions that were analyzed by electrospray ionization mass spectroscopy (ESI-MS) and to unravel the solution processes that unfold on the column to explain the observed retention behavior. These solution processes are potentially relevant to understanding the disposition/excretion of these mercurials via known molecular transport pumps, such as multidrug-resistant protein-2 (MRP2) in the liver [31] and organic anion transporter-1 (OAT1) in the kidneys [32].

## 2. Results and Discussion

**Figure 2:**
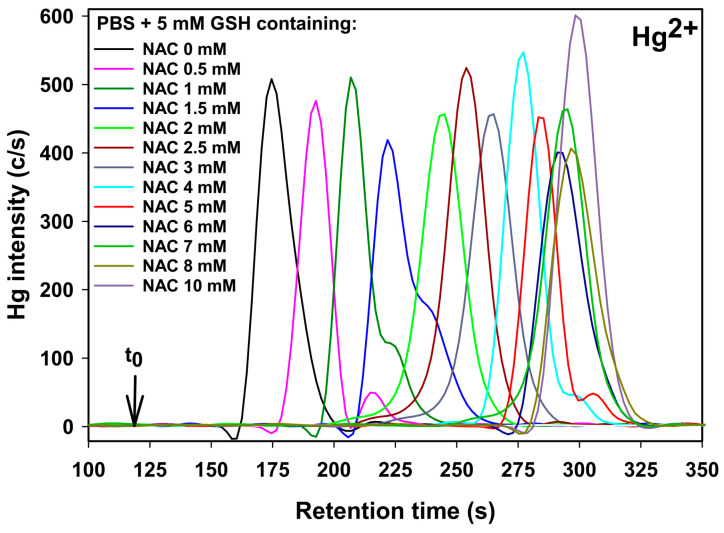
Representative Hg-specific chromatograms obtained after the injection of Hg^2+^ on a Gemini C_18_ RP-HPLC column (150 × 4.6 mm I.D.) at 37 °C using 5.0 mM GSH with 0–10 mM NAC in PBS buffer (pH 7.4). Flow rate: 1.0 mL/min. Detector: ICP-AES (Hg at 253.65 nm). Injection volume: 20 µL (5 μg Hg).

**Figure 3:**
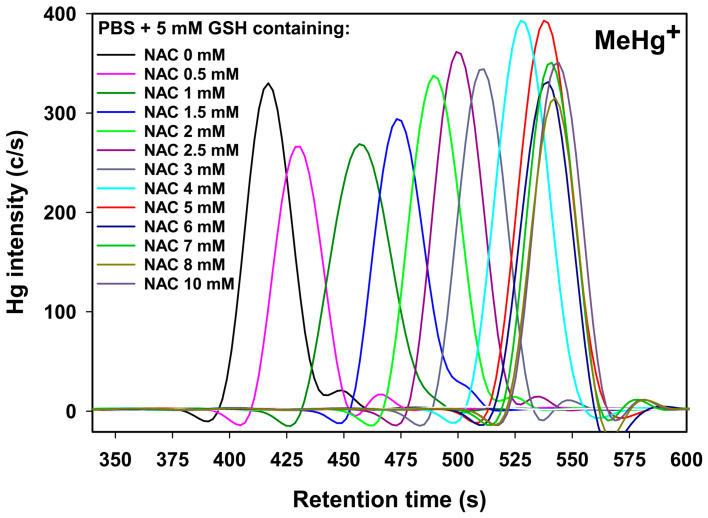
Representative Hg-specific chromatograms obtained after the injection of MeHg^+^ on a Gemini C_18_ RP-HPLC column (150 × 4.6 mm I.D.) at 37 °C using 5.0 mM GSH with 0–10 mM NAC in PBS buffer (pH 7.4). Flow rate: 1.0 mL/min. Detector: ICP-AES (Hg at 253.65 nm). Injection volume: 20 µL (5 μg Hg).

**Figure 4:**
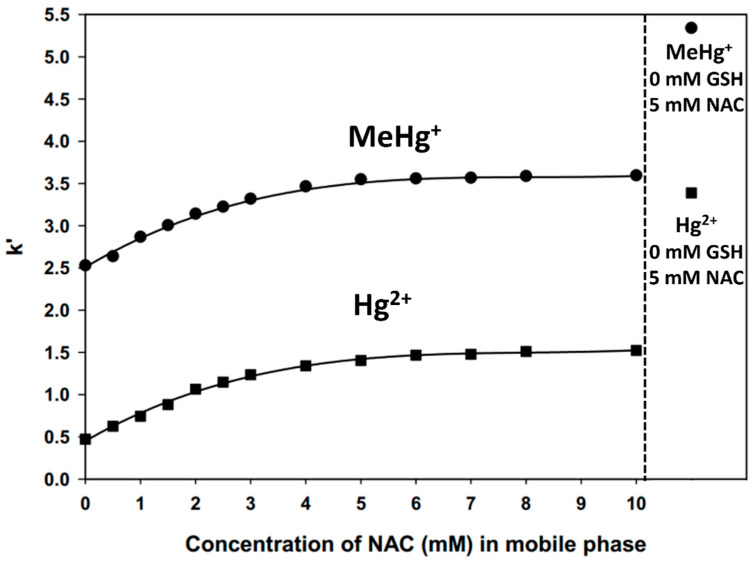
Retention behavior of Hg^2+^ and MeHg^+^ on a Gemini C_18_ RP-HPLC column (150 × 4.6 mm I.D.) at 37 °C. Mobile phase: 5.0 mM GSH with 0–10 mM NAC in PBS buffer (pH 7.4). Flow rate: 1.0 mL/min. Detector: ICP-AES (Hg at 253.65 nm). Injection volume: 20 μL (5 μg Hg). Symbols on the right side correspond to the k′-value of each metal species using PBS buffer with 5.0 mM NAC as the mobile phase (GSH-free).

The biochemical mechanisms that determine the fate of Hg^2+^ and MeHg^+^ after they enter mammalian cells are incompletely understood [33,34], and very little is known about the mechanisms that are involved in their urinary excretion [10]. Therefore, research focused on the bioinorganic processes governing their intracellular trafficking holds considerable toxicological importance [35]. To this end, PBS buffer mobile phases, which contained 5.0 mM GSH and increasing NAC concentrations (0.5–10.0 mM), were used to systematically investigate the retention behavior of Hg^2+^ and MeHg^+^ on a C_18_ RP-HPLC column. The obtained Hg-specific chromatograms are depicted in Figure 2 and Figure 3. To better visualize the differences in the retention behavior of these mercurials, the retention factors (*k*′-values) that were obtained for the main Hg-peak of each mercurial were plotted as a function of the NAC concentration in the mobile phase (Figure 4).

**Figure 5:**
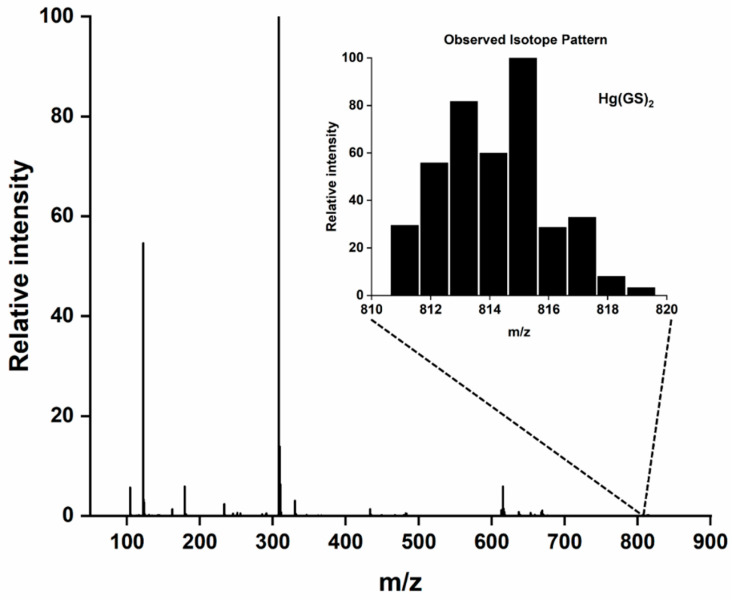
ESI-MS identification of the Hg^2+^ complex after RP-HPLC-ICP-AES analysis using 50 mM Tris buffer (pH 7.4, 5.0 mM GSH) as the mobile phase.

With the 5.0 mM GSH mobile phase, the injection of Hg^2+^ produced a single Hg peak that displayed tailing (Figure 2). The marginal retention of this Hg-peak on the C_18_ stationary phase (*k*′ = 0.47) aligns well with previous results obtained on the same RP-HPLC column [30]. This behavior can be attributed to the on-column formation of Hg(GS)_2_ and Hg(GS)_3_ complexes [35], which weakly interact with the C_18_ groups of the stationary phase due to the hydrophobic methylene moieties of GSH being rather buried within its structure (Figure 1). To identify the Hg complexes formed on the column, the experiment was repeated with a mobile phase containing 5.0 mM GSH in 50 mM Tris buffer (pH 7.4). Upon injecting Hg^2+^, a single Hg-peak with a similar retention time to that observed with the corresponding PBS buffer mobile phase was obtained . The ESI-MS analysis of the collected Hg-peak confirmed the on-column formation of Hg(GS)_2_, as evidenced by the expected Hg isotope pattern (Table 1, Figure 5), which is consistent with previous results that revealed di-thiolate binding of Hg^2+^ in multi-cysteinyl peptides [36]. Since Hg(GS)_3_ species are known to form in solutions with excess GSH at physiological pH [35] and in cyclic decapeptides [37], it is likely that a mixture of Hg(GS)_2_ and Hg(GS)_3_ was eluted from the column.

**Figure 6:**
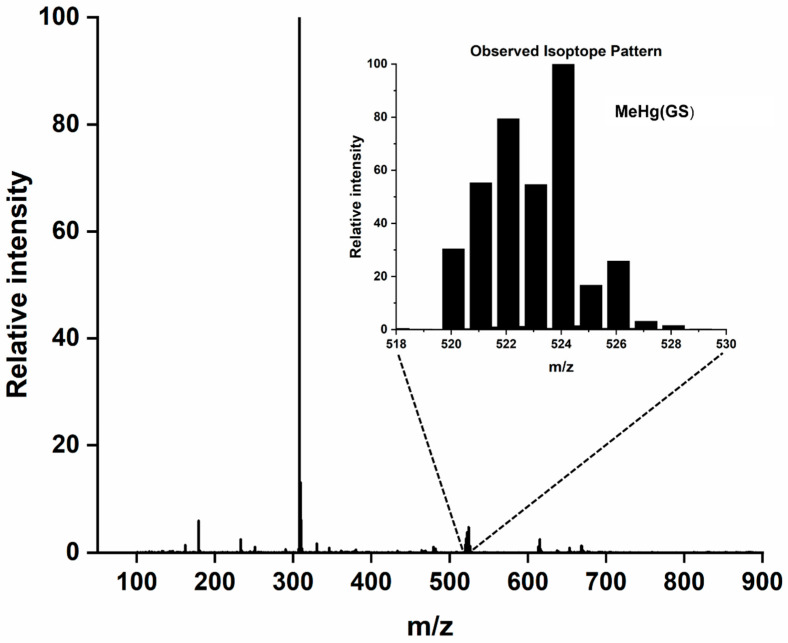
ESI-MS identification of the MeHg^+^ complex after RP-HPLC-ICP-AES analysis using 50 mM Tris buffer (pH 7.4, 5.0 mM GSH) as the mobile phase.

When MeHg^+^ was injected, it was observed to be more strongly retained (*k*′ = 3.80) compared to Hg^2+^ (*k*′ = 0.47), which is in good agreement with previous results obtained using the same C-_18_ RP-HPLC stationary phase [30]. Owing to the high affinity of MeHg^+^ for thiols, a MeHg(GS) complex was likely formed on the on-column, which resulted in a comparatively stronger interaction with the C_18_ groups of the stationary phase. To corroborate the on-column formation of MeHg(GS) complexes, the experiment was repeated with a mobile phase that contained 5.0 mM GSH in 50 mM Tris buffer (pH 7.4), which revealed a Hg-peak with a similar retention time as the one observed with the corresponding PBS buffer mobile phase. The analysis of the Hg-peak by ESI-MS (Table 1, Figure 6) confirmed the on-column formation of a MeHg(GS) complex. 

### 2.1. Retention Behavior of Hg^2+^ as a Function of the NAC Mobile Phase Concentration

To enhance the clarity of the discussion of the retention behavior, we will initially focus on the results obtained for mobile phases with 5.0 mM GSH and NAC concentrations up to 5.0 mM and then discuss the results achieved for mobile phases containing 6.0–10.0 mM NAC.

**Scheme 1:**
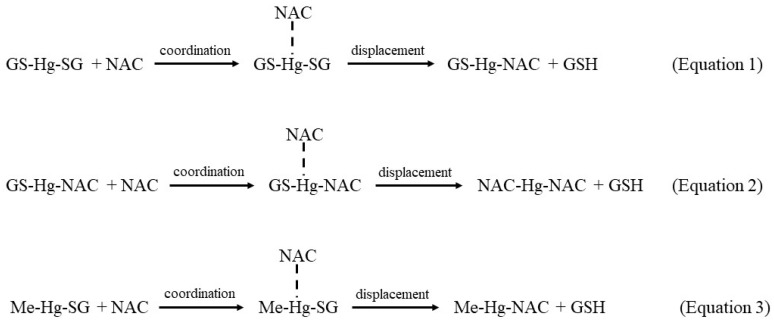
Illustration of the proposed reactions of Hg^2+^ and MeHg^+^ with GSH and NAC in the mobile phase that can help to explain their retention behavior observed by SEC-ICP-AES.

When the NAC mobile phase concentration was elevated from 0.5 mM to 5.0 mM, the retention times of the main Hg-peak gradually extended from 192 s to 284 s (Figure 2) and the corresponding *k*′-values increased from 1.06 to 1.40 (Figure 4). Since the GSH concentration was constant in all experiments, the obtained results imply the on-column formation of progressively more hydrophobic Hg^2+^-GS_x_-NAC_y_ species that more strongly interacted with the C_18_-stationary phase. Interestingly, varying NAC concentrations in the mobile phases (0.5, 1.0, 1.5, 4.0, and 5.0 mM) resulted in an interesting chromatographic elution pattern. Specifically, an intense Hg-peak was followed by a much smaller peak on its long retention end (Figure 2), which implies the on-column formation of different types of Hg species. The gradual increase in the retention time of the observed Hg peaks can be rationalized by two processes. The first process, referred to as the ‘coordination process’, involves the coordination of NAC via its thiol group to the Hg-center of the linear species Hg(GS)_2_ (Scheme 1, Equation (1), middle). This explanation is based on the observation that the third ligand in Hg(GS)_3_ is bound more weakly than the first two [38] and that the formation constant for the Hg(NAC)_2_ complex is slightly larger than that for Hg(GS)_2_ [12,39]. This coordination results in the formation of a tri-coordinate Hg-species [39,40,41], which exhibits a higher hydrophobicity compared to Hg(GS)_2_/Hg(GS)_3_ and will therefore be more strongly retained by the C_18_-groups of the stationary phase, leading to longer retention times. The second process, referred to as the ‘displacement process’, entails the displacement of a GS-moiety that is bound to the Hg-center by the coordinated NAC (Scheme 1, Equation (1), right).

This process can then be repeated at higher NAC concentrations (Scheme 1, Equation (2)), resulting in progressively more hydrophobic Hg complexes. The ‘coordination process’ predominantly occurs at lower NAC mobile phase concentrations, while the ‘displacement process’ dominates at higher concentrations. As the NAC concentration is increased, both of these processes will sequentially take place in solution, making it challenging to distinguish them solely based on the obtained Hg-specific chromatograms. Therefore, ESI-MS was employed to study the nature of Hg-species that eluted from the column when using a 5.0 mM NAC in the 5.0 mM GSH mobile phase.

To understand the retention behavior of Hg^2+^ across the 0.5–5.0 mM NAC range, in terms of its chemistry with both thiols on the column, it is instructive to reiterate that with the 5.0 mM GSH mobile phase, relatively hydrophilic Hg(GS)_2_ and Hg(GS)_3_ species were formed (Table 1, Figure 5).

**Figure 7:**
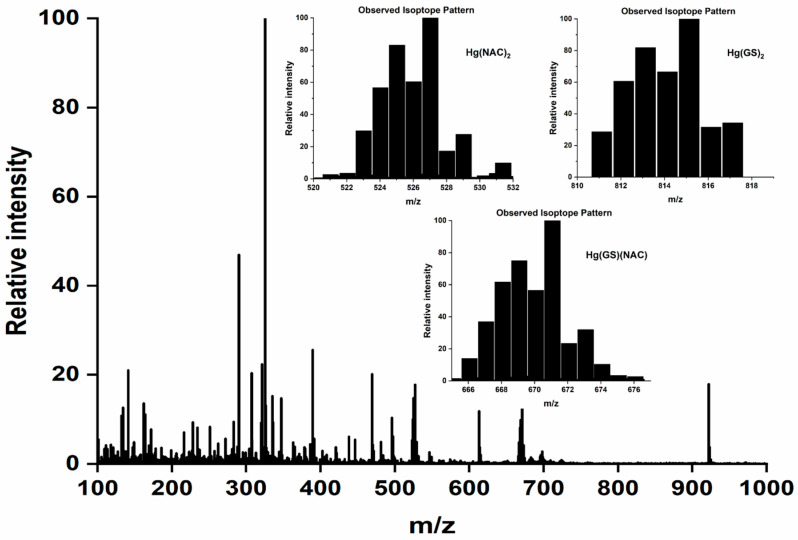
ESI-MS identification of the Hg^2+^ complexes Hg(NAC)_2_, Hg(GS)(NAC), and Hg(GS)_2_ after RP-HPLC-ICP-AES analysis using 50 mM Tris buffer (pH 7.4, 5.0 mM GSH, 5.0 mM NAC) as the mobile phase.

Since the upward trend in the retention time of Hg^2+^ with increasing NAC concentrations did not continue beyond 5.0 mM, we investigated the Hg-species that eluted from the column at this NAC concentration in the mobile phase. Therefore, Hg^2+^ was injected using a mobile phase that contained 5.0 mM GSH and 5.0 mM NAC in 50 mM Tris buffer (pH 7.4) and the collected Hg-peak was analyzed by ESI-MS. The results revealed a mixture of Hg(GS)(NAC)(49.8%), Hg(NAC)_2_ (37.5%), and Hg(GS)_2_ (12.7%) (Figure 7). While the presence of ~50% of a mixed Hg(GS)(NAC) species is expected given that the mobile phase contained equimolar concentrations of GSH and NAC (i.e., 5.0 mM), the formation of a considerable fraction of Hg(NAC)_2_ implies a higher comparative affinity of Hg^2+^ for NAC than GSH under the investigated experimental conditions. Based on these ESI-MS results and assuming that the Tris buffer does not appreciably affect the solution chemistry, the retention behavior of Hg^2+^ between 0.5 and 5.0 mM NAC can be rationalized by a combination of the aforementioned ‘coordination’ and ‘displacement’ processes of NAC at the Hg(GS)_2_ species (Scheme 1, Equations (1) and (2)). Accordingly, the addition of 0.5–2.0 mM NAC to the mobile phase progressively increased the retention of the major Hg-peak due to the ‘coordination process’, leading to the on-column formation of a more hydrophobic NAC^…^Hg(GS)_2_ species (Scheme 1, Equation (1), left). Simultaneously, a minor Hg-peak, exhibiting a significant increase in the intensity over this concentration range may be tentatively attributed to the ‘displacement process’ which is associated with the on-column formation of Hg(GS)(NAC) (Scheme 1, Equation (1), right). Hence, the single Hg-peak that was observed with the 2.0 mM NAC mobile phase may be largely comprised of Hg(GS)(NAC). With a further increase in the NAC concentration from 2.5 mM to 5.0 mM, there was a progressive augmentation of the retention time of the major Hg-peak, resulting from another ‘coordination process’. This process involved the formation of an even more hydrophobic species NAC^…^Hg(GS)(NAC) (Scheme 1, Equation (2), left), followed by the formation of the ‘displacement species’ Hg(NAC)_2_. This interpretation is in general accord with the presence of Hg(GS)(NAC) and Hg(NAC)_2_ when using a 5.0 mM NAC (Table 1, Figure 7). Taken together, the observed chromatographic retention behavior of Hg^2+^ when combined with ESI-MS analysis allowed us to obtain a reasonable explanation of the underlying solution chemistry processes pertaining to the competitive interaction of Hg^2+^ with GSH and NAC.

With regard to the 6.0–10.0 mM NAC concentration range, no significant further increase in the *k*′-values of the Hg-peaks was observed (*k*′ at 6.0 mM = 1.46, *k*′ at 10.0 mM = 1.52). This chromatographic retention behavior can be explained by the fact that the hydrophobicity of the on-column formed Hg(NAC)_2_ (which already constituted 37.5% of the total Hg with the 5.0 mM NAC mobile phase) reaches a limit and can no longer be increased. This limitation is likely due to the presence of 5.0 mM GSH, which results in a considerable fraction of Hg^2+^ being complexed as Hg(GS)(NAC), thus precluding the formation of NAC^…^Hg(NAC)_2_, and Hg(NAC)_3_. Owing to the limited physiological relevance of mobile phases, which contain >5.0 mM NAC, column fractions were not analyzed by ESI-MS.

To corroborate the biomolecular explanation for the observed chromatographic retention behavior of Hg^2+^, a chromatographic control experiment was conducted, using a GSH-free mobile phase containing 5.0 mM NAC in PBS buffer (Figure 4, right). The obtained results revealed a *k*′-value of 3.38, which was ~2.4 fold larger than the value obtained for 5.0 mM NAC with 5.0 mM GSH (*k*′ = 1.40). This result is rationalized by the on-column formation of a mixture of Hg(NAC)_3_ and Hg(NAC)_4_ complexes [39], which—owing to the presence of their intrinsically hydrophobic acetyl-moieties—are considerably more hydrophobic than the Hg(GS)(NAC) and Hg(NAC)_2_ that are predominantly formed in a 5.0 mM NAC and 5.0 mM GSH mobile phase (Table 1, Figure 7).

### 2.2. Retention Behavior of MeHg^+^ as a Function of the NAC Mobile Phase Concentration

To improve the clarity of the discussion regarding the observed retention behavior, we will first focus on the results obtained within the NAC mobile phase concentration range up to 5.0 mM and then elaborate on the results obtained for the 6.0-10.0 mM NAC range. 

**Figure 8:**
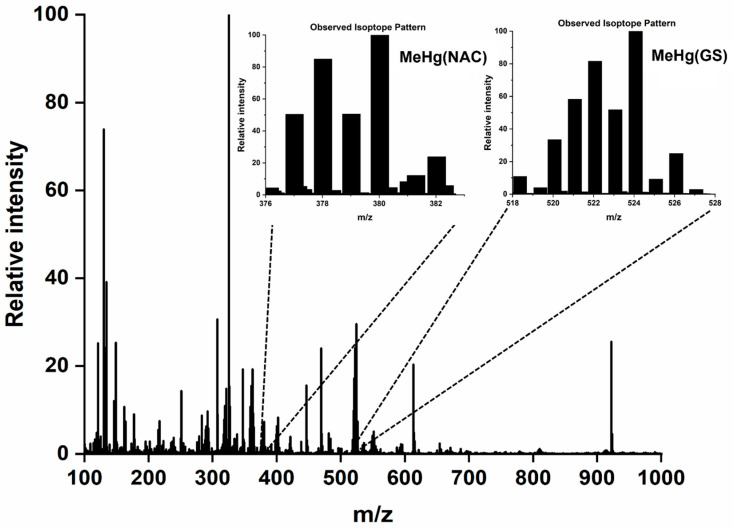
ESI-MS identification of the CH_3_Hg^+^ complexes CH_3_Hg(NAC) and CH_3_Hg(GS) after RP-HPLC-ICP-AES analysis using 50 mM Tris buffer (pH 7.4, 5.0 mM GSH, 5.0 mM NAC) as the mobile phase.

When the NAC concentration in the mobile phase was varied from 0.5 to 5.0 mM, the retention time of the single Hg-peak increased from 430 to 537 s (Figure 3), and the corresponding *k*′-values steadily rose from 2.53 to 3.54. This retention behavior can be attributed to two possible processes: the ‘coordination process’ leading to the formation of a progressively more hydrophobic NAC^…^MeHg(GS) species and/or the ‘displacement process’ resulting in the formation of MeHg(NAC) (Scheme 1, Equation (3)) [39]. To identify the Hg-complex responsible for the observed retention behavior, a follow-up experiment was conducted using 5.0 mM GSH and 5.0 mM NAC in 50 mM Tris buffer (pH 7.4). The obtained Hg-peak was collected, and ESI-MS results (Table 1, Figure 8) revealed the presence of a mixture of MeHg(GS) (98.7%) and MeHg(NAC) (1.31%). While these ESI-MS results may not appear to align with the proposed sequential ‘coordination process’ and ‘displacement process’, it is crucial to consider that the coordination species NAC^…^MeHg(GS) [and the putative NAC^…^MeHg(NAC)] may not exhibit sufficient stability to produce a detectable signal in the ESI-MS. Thus, the detected MeHg(GS) species may actually correspond to the coordinative species NAC^…^MeHg(GS), which did not survive the ionization process.

To validate the aforementioned biomolecular explanation for the chromatographic retention behavior of MeHg^+^, a control experiment was conducted using a 5.0 mM NAC mobile phase (Figure 4, right). The observed *k*′-value of MeHg^+^ was measured to be 5.34, which is ~1.5 fold larger than the corresponding value obtained for the 5 mM NAC with 5.0 mM GSH mobile phase (*k*′ = 3.54). This significant increase in MeHg^+^ retention can be rationalized in terms of the exclusive formation of the comparatively more hydrophobic MeHg(NAC) in the GSH-free mobile phase. This observation suggests that with the 5 mM GSH and 5 mM NAC mobile phases in PBS buffer, a MeHg species was eluted from the column that coordinated with both thiols equally. Between 6.0–10.0 mM NAC in the 5.0 mM GSH mobile phase, only a negligible increase in the *k*′ was observed (3.56 vs. 3.59), which was explained by the fact that the hydrophobicity of the formed MeHg species (i.e., MeHg(NAC)) does not increase as the MeHg undergoes rapid exchange with other NAC species in the mobile phase.

### 2.3. Comparison of NAC Results to Cys-Related Chromatographic Results

In a previous study, the impact of introducing Cys (0.5–10 mM) as a competing thiol to the 5.0 mM GSH-containing mobile phase on the same C_18_ RP-HPLC column was investigated. The results revealed decreasing retention times for Hg^2+^ and MeHg^+^, which indicated the formation of progressively *less* hydrophobic species compared to the 5.0 mM GSH mobile phase [30]. Notably, the retention time of MeHg^+^ decreased gradually, while the retention time of Hg^2+^ initially remained unaffected by Cys but then smoothly decreased to a first and eventually a second plateau. The differences in the retention behavior of these mercurials were attributed to the replacement of hydrophobic GS-moieties (coordinated to each mercurial) by comparatively less hydrophobic Cys-moieties (log *K* for Hg(Cys)_2_ = 10^15^–10^42^ [42] vs. Hg(GS)_2_ = 10^42^) [12] and differences in the stability of Hg-S bonds of the on-column formed complexes.

In contrast, the utilization of NAC as the competing thiol resulted in a progressive rise in the retention times of both mercurials. These observations suggested the formation of *more* hydrophobic Hg-complexes, likely containing bound NAC, which is comparatively more hydrophobic than GSH (Figure 1). Importantly, it should be highlighted that introducing NAC to the 5.0 mM GSH mobile phase resulted in a gradual increase in the retention time for both mercurials. This observed trend is congruent only with the previously recorded retention behavior of MeHg^+^ upon the addition of Cys to the 5.0 mM GSH mobile phase, but different from the corresponding retention behavior of Hg^2+^ [30]. The aforementioned retention behavior for both mercurials can be rationalized by the coordination of NAC with the on-column generated Hg(GS)_2_, distorting its linear GS-Hg-SG structure and that of MeHg(GS) species to form tri-coordinate species, which can better interact with the C_18_ groups of the stationary phase, resulting in greater hydrophobic characteristics (Scheme 1). Given that an evidently larger retention time of the on-column formed mercurials was noticeable even at the introduction of 0.5 mM NAC in the 5.0 mM GSH mobile phase (Figure 2, Figure 3 and Figure 4), it is plausible that the formation of the corresponding tri-coordinate species at near-physiological conditions could play a role in the small molecular weight thiol-mediated intracellular disposition of these mercurials to different sub-cellular compartments within mammalian cells [43] as well as their biliary excretion [25].

### 2.4. Potential Bioinorganic Implications of the Findings

Our results offer novel insight into fundamental processes that may explain certain outcomes from previous animal experiments where concurrent exposure to either mercurials or NAC was investigated [10,44]. For example, it was observed in rats which had been given MeHg^+^, that this mercurial could be efficiently mobilized from the tissues when NAC was administered in the drinking water [26]. Our results suggest that these observations could be attributed to the experimentally observed displacement of GS-moieties from MeHg^+^ within the liver cytosol (and possibly other organ cells) as a consequence of the orally administered NAC, followed by the transfer of the intracellularly formed MeHg(NAC) to the MRP-2 transporter, which facilitates its rapid excretion from the hepatocyte to the bile [31]. Furthermore, it is noteworthy that MeHg(NAC) has been identified as a substrate for the OAT1 transporter [26,32], which is expressed in the kidneys [45]. This particular MeHg(NAC)-species has been shown to be taken up in vivo at the basolateral membrane of proximal tubular epithelial cells [26], subsequently leading to its excretion from the kidneys via the urine. Thus, even though the formation of MeHg(NAC) was observed at near-physiological conditions of the cytosol in the absence of proteins, the presence of MRP-2 and OAT1 in excretory organs suggests that our results are of potential toxicological significance.

The effect of NAC on the toxicology of Hg^2+^ has also been investigated in animal models; however, the results were ambiguous, as experiments with rats have demonstrated an amelioration of its toxicity through NAC administration [46], whereas experiments with mice have revealed a potentiation of the toxicity of Hg^2+^ by NAC [47]. Related to these findings, NAC has also been shown to ameliorate the toxicity of other toxic metal species, such as Cd^2+^ [48], as well as the metal-based drug cis-platin in animal models [49,50]. Thus, the developed liquid chromatographic approach holds promise to gain important new insight into bioinorganic processes within the cell cytosol which critically determine either the disposition of toxic metal species to subcellular compartments and/or their excretion from the cell. The developed approach is useful to extend investigations into the molecular toxicology of metal(loid)s beyond the bloodstream [51] into organs and may also prove valuable in the development of nutraceuticals to ameliorate the toxic effects of inorganic pollutants in affected populations [52], as recent studies have shown that different molecular Hg forms are prevalent in human brain tissues depending on the exposure dose [8].

## 3. Materials and Methods

HgCl_2_ and CH_3_Hg^+^ are highly toxic compounds. All sample preparation procedures involved wearing disposable Latex gloves and conducting the necessary experiments in a ventilated laboratory fume hood.

### 3.1. Chemicals

Regarding chemicals, 0.01 M phosphate buffered saline (PBS) powder sachets (0.01 M NaH_2_PO_4_, 0.138 M NaCl, 0.0027 M KCl), Tris(hydroxymethyl)-aminomethane (Trizma base, ≥99.9%), sodium chloride (NaCl ≥ 99.5%), sodium hydroxide pellets (NaOH, ≥97%), mercuric chloride (HgCl_2_, ≥99.5%), L-cysteine (Cys, ≥98% purity), and L-glutathione reduced (GSH, ≥98% purity) were purchased from Sigma-Aldrich (St. Louis, MO, USA). HCl (plasma pure 34–37%) was purchased from SCP Science (Baie D’Urfe, QC, Canada), while CH_3_HgOH (aqueous 1.0 M solution) was obtained from Alfa Aesar (Haverhill, MA, USA). Arsenobetaine bromide (AB) was synthesized according to a previously published procedure, and its recrystallization from ethanol yielded white crystals with a melting point of 225 °C (literature: 227 °C) [53].

### 3.2. Solutions and Mobile Phases

All solutions and mobile phases were prepared using deionized water from a Simplicity UV water purification system (18.2 MΩ cm, Millipore, Billerica, MA, USA). Aqueous solutions of AB (3.3 mM) and HgCl_2_ (1.3 mM) were prepared by dissolving each compound in deionized water. A solution of CH_3_HgOH (1.3 mM) was prepared by diluting an aqueous 1.0 M CH_3_HgOH solution with deionized water. The AB solution contained 5 μg As/20 μL (injection volume), while each mercurial solution contained 5 μg Hg/20 μL.

The mobile phases were prepared using PBS buffer, which was obtained by dissolving PBS buffer sachets in the appropriate volume of deionized water. An appropriate amount of GSH (5.0 mM) and/or NAC (0.5–10 mM) was accurately weighed and dissolved in PBS buffer before the pH was subsequently adjusted to 7.4 by dropwise addition of HCl/NaOH, if necessary. In order to reduce the amount of dissolved oxygen in the buffer and thus the oxidation of any thiol groups in the solution, all thiol-containing solutions were prepared fresh prior to each chromatographic run and then subjected to N_2_(g) bubbling throughout the entire experiment. All pH measurements were performed with a VWR Symphony SB20 pH meter (Thermo Electron Corporation, Beverly, MA, USA). Prior to use, all solutions were filtered through MF-Millipore 0.45 μm MCE Nitrocellulose Membranes (Mandel Scientific, Guelph, ON, Canada).

### 3.3. RP-HPLC-ICP-AES

The RP-HPLC system was comprised of an Agilent 1200 series binary pump high-performance liquid chromatography (HPLC) pump and a Rheodyne 9725 injector equipped with a 20 μL PEEK sample loop and a Gemini RP column (150 × 4.6 mm I.D., 5.0 m particle size; in conjunction with a 4 × 3 mm I.D. guard cartridge, Phenomenex, Torrance, CA, USA). The Gemini stationary phase is a silica/polymer hybrid that combines the chemical inertness of a polymer with the mechanical strength and efficiency of silica, resulting in extended pH stability (pH 1–12). All chromatographic investigations were carried out at 37 °C using a Thermasphere™ TS-130 HPLC column temperature controller (Phenomenex; Torrance, CA, USA). The column was equilibrated with at least 60 mL of mobile phase before the injection of Hg compounds, and the flow rate was maintained at 1.0 mL/min. The dead time (t_0_) of the column was determined by injecting a NaCl aqueous solution (14 mM) using deionized water as the mobile phase. The emission of Na was detected at t_0_ of 118 s. To assess the integrity of the HPLC column throughout the study, a 20 μL aliquot of an aqueous solution of AsB (3.3 mM) was injected after every second mobile phase. AsB does not chemically interact with thiols from the mobile phase, but it is retained on the column owing to the interaction of its methyl groups with the stationary phase. The retention time of AsB remained consistent regardless of the mobile phase composition, which represents proof that the injection of the Hg solutions did not compromise the HPLC column’s integrity. As and Hg were detected in the column effluent with a Prodigy, high dispersion, radial view ICP AES (Teledyne-Leeman Labs, Hudson, NH, USA) with the following parameters: a radio frequency (RF) power of 1.3 kW, an Ar gas coolant flow rate of 19 L/min, an auxiliary flow rate of 0.5 L/min, and a nebulizer gas pressure of 25 psi. As was monitored at 189.042 nm, and Hg was at 253.65 nm. All injected metal species produced peaks, and the raw data collected using the ICP-AES data acquisition and controller software (SALSA, Version 3.0) were imported into Sigma Plot 14.5 (Chicago, IL, USA) and smoothed using the bisquare algorithm. The retention times were calculated based on triplicate injections (RSD ≤ 1.6). To better visualize the comparative chromatographic results that were obtained for Hg^2+^ and MeHg^+^ compared to previous work, the retention times for the main Hg-peak were converted to *k*′-values by subtracting the retention time of the unretained NaCl species (i.e., t_0_) from the actual retention time and dividing the obtained number by t_0_.

### 3.4. Electrospray Ionization Mass Spectrometry (ESI-MS)

To identify the Hg-species that were eluted with the NAC and GSH-containing PBS buffer mobile phases, each mercurial was injected into the Gemini RP column using 50 mM Tris buffer containing either 5.0 mM GSH or 5.0 mM GSH with 5.0 mM NAC (pH 7.4). The Hg-specific chromatograms were used to determine the retention times of the on-column-formed complexes. Then another aliquot of each mercurial was injected, and the centers of Hg^2+^ and MeHg^+^-containing peak fractions (60 s width) were collected and analyzed by LC-ESI-MS. The latter analysis was performed using an Agilent 1200 series HPLC coupled to an Agilent 6520 Q-TOF mass spectrometer. Partial separation of the Hg complexes from the buffer matrix was achieved using a Zorbax Eclipse Plus C_18_ column (2.1 mm × 50 mm, 1.8 um particle size) and a 0.1% formic acid in water/methanol gradient (0–1 min 20% MeOH, 1–9 min gradient to 90% MeOH, 9–10 min 90% MeOH). The injection volume was 1.0 uL. Extracted ion chromatograms (EIC) of the Hg complexes were obtained from the TIC using the calculated theoretical mass of ± 10 ppm. The total peak areas that were obtained for Hg^2+^ and MeHg^+^ were 227,361 and 221,508, respectively, which implies that similar quantities of both mercurials reached the MS and the results are therefore comparable.

## 4. Conclusions

The interaction of toxic metal species, such as Hg^2+^ and MeHg^+^, with multiple cytosolic ligands within mammalian cells, is pivotal in shaping their toxicological fate [30] as well as their biliary excretion [54]. The underlying processes that unfold at the corresponding physiological conditions are incompletely understood. To shed light on the competitive interaction of Hg^2+^ and MeHg^+^ with GSH and the small molecular weight thiol ligand NAC at near-physiological conditions, their retention behavior was investigated on a C_18_ RP-HPLC column using a 5.0 mM GSH-containing mobile phase (PBS buffer) with 0.5–10.0 mM NAC. The gradual increase in the hydrophobicity of the on-column-formed mercury complexes with increasing NAC concentrations was rationalized in terms of the competitive interaction of each mercurial with GSH and NAC to form complexes that subsequently interact with the C_18_-groups of the stationary phase. With regard to Hg^2+^, the analysis of Hg-containing fractions obtained with 50 mM Tris buffer at 5.0 mM GSH and NAC by ESI-MS allowed us to observe the predominant formation of Hg(GS)(NAC) followed by Hg(NAC)_2_, while for MeHg^+^, the prevalent species that was detected was MeHg(GS), followed by MeHg(NAC) as a minor species. The chromatographic retention behavior of both mercurials was rationalized by a progressive NAC-mediated coordination to the Hg-center of Hg(GS)_2_ and MeHg(GS) followed by the eventual displacement of the GS-moieties from Hg^2+^ and MeHg^+^ to form the more hydrophobic Hg-species Hg(GS)(NAC) and Hg(NAC)_2_ as well as MeHg(NAC) on the column. These processes could also potentially unfold within hepatocytes and are therefore of toxicological relevance. Our findings demonstrate that the application of RP-HPLC-ICP-AES in conjunction with ESI-MS represents a versatile bioanalytical method to investigate competitive interactions that unfold between toxic metal species with multiple thiol ligands under near-physiological conditions. While the obtained results provide insight into important solution chemistry-based processes that may unfold in any given cell cytosol, knowledge about the interaction of the formed metal-ligand complexes with molecular export mechanisms that are located at the hepatocyte/bile junction [55] and/or the kidneys are absolutely critical in order to evaluate if the uncovered processes are possibly involved in the toxicologically important excretion of the involved metal species from mammals.

## Figures and Tables

**Table 1:** ESI-MS identification of Hg-species that eluted from the RP-HPLC column.

Mobile Phase Composition	Identified Hg Species/Formula	Theoretical Mass of the Most Abundant Isotope (Hg^202^) Da	Experimental Mass of the Most Abundant Isotope (Hg^202^) Da	ΔM_theor-exp_ Da
5.0 mM GSH	Hg(GS)_2_ C_20_H_32_N_6_O_12_S_2_	815.1299	815.1290	0.0009
5.0 mM GSH and 5.0 mM NAC	Hg(NAC)_2_ C_10_H_16_N_2_O_6_S_2_	527.0229	527.0226	0.003
	Hg(GS)_2_ C_20_H_32_N_6_O_12_S_2_	815.1299	815.1318	−0.0001
	Hg(NAC)(GS) C_15_H_24_N_4_O_9_S_2_	671.0764	671.0750	0.0014
5.0 mM GSH	MeHg(GS) C_11_H_19_N_3_O_6_S_1_	524.0774	524.0774	0.0005
5.0 mM GSH and 5.0 mM NAC	MeHg(GS) C_11_H_19_N_3_O_6_S_1_	524.0774	524.0759	0.0015
	MeHg(NAC) C_6_H_11_N_1_O_3_S_1_	380.0239	380.0227	0.0012

## Data Availability

All data are contained within the article.
